# Logistic burdens of cancer care: A qualitative study

**DOI:** 10.1371/journal.pone.0300852

**Published:** 2024-04-04

**Authors:** Allison C. Dona, Patricia I. Jewett, Sharon Hwee, Katherine Brown, Matia Solomon, Arjun Gupta, Deanna Teoh, Guang Yang, Julian Wolfson, Yingling Fan, Anne H. Blaes, Rachel I. Vogel

**Affiliations:** 1 School of Medicine, University of Minnesota, Minneapolis, Minnesota, United States of America; 2 Division of Epidemiology and Community Health, University of Minnesota, Minneapolis, Minnesota, United States of America; 3 Department of Obstetrics, Gynecology, and Women’s Health, University of Minnesota, Minneapolis, Minnesota, United States of America; 4 Division of Hematology, Oncology and Transplantation, University of Minnesota, Minneapolis, Minnesota, United States of America; 5 Division of Pediatric Hematology and Oncology, University of Minnesota, Minneapolis, Minnesota, United States of America; 6 Daynamica, Inc., Chanhassen, Minnesota, United States of America; 7 Division of Biostatistics and Health Data Science, University of Minnesota, Minneapolis, Minnesota, United States of America; 8 Humphrey School of Public Affairs, University of Minnesota, Minneapolis, Minnesota, United States of America; University of Birmingham, UNITED KINGDOM

## Abstract

Cancer treatment often creates logistic conflicts with everyday life priorities; however, these challenges and how they are subjectively experienced have been largely unaddressed in cancer care. Our goal was to describe time and logistic requirements of cancer care and whether and how they interfered with daily life and well-being. We conducted interviews with 20 adults receiving cancer-directed treatment at a single academic cancer center. We focused on participants’ perception of the time, effort, and energy-intensiveness of cancer care activities, organization of care requirements, and preferences in how to manage the logistic burdens of their cancer care. Participant interview transcripts were analyzed using an inductive thematic analysis approach. Burdens related to travel, appointment schedules, healthcare system navigation, and consequences for relationships had roots both at the system-level (e.g. labs that were chronically delayed, protocol-centered rather than patient-centered bureaucratic requirements) and in individual circumstances (e.g. greater stressors among those working and/or have young children versus those who are retired) that determined subjective burdensomeness, which was highest among patients who experienced multiple sources of burdens simultaneously. Our study illustrates how objective burdens of cancer care translate into subjective burden depending on patient circumstances, emphasizing that to study burdens of care, an exclusive focus on objective measures does not capture the complexity of these issues. The complex interplay between healthcare system factors and individual circumstances points to clinical opportunities, for example helping patients to find ways to meet work and childcare requirements while receiving care.

## Introduction

Cancer treatment is often time-intensive. Beyond the time of getting treatment itself, treatment-related logistic burden encompasses scheduling and attending appointments, pharmacy visits, consultations, insurance paperwork, managing drugs, transportation, and wait time. Balancing cancer treatment tasks and side effects with everyday life activities can create numerous logistic and administrative challenges for those living with cancer [1].

In this manuscript, we refer to the everyday challenges of carrying out cancer care activities as the “logistic toxicity” of cancer care [2]. Coined in a non-academic piece in 2015 [3], logistic toxicity is familiar to many patients, but the study of logistic toxicity of cancer is in its early stages. Logistic toxicity is similar and conceptually related to another aspect of cancer treatment burden: the “financial toxicity” of cancer, defined as the cumulative financial impact of cancer and its treatments on patients [4–6]. Many patients undergoing cancer care experience large material costs and subsequent emotional distress due to financial strain [7, 8] such that financial toxicity of cancer plays out on multi-dimensional (objective and subjective) domains [9]. Analogously, effects of logistic toxicity may be objective (time and number of cancer-related tasks), and context-dependent or subjective, i.e., biographical (e.g. time opportunity costs, loss of independence), relational (familial stress and strained social relationships), and psychosocial (anxiety, stress, anger, worry, etc.) [10]. Support persons may also experience logistic burdens of cancer, as caregivers often accompany patients to clinic visits for treatment or side effect management [11]. Ultimately, logistic toxicity may affect treatment access, effectiveness, and outcomes [10, 12–19] as well as compound systemic burdens for disadvantaged patients [20–24].

Measuring the contributors to and consequences of logistic toxicity poses multiple challenges. While the number of appointments, travel time, and wait time for visits to healthcare facilities can be objectively recorded [25–30], context-dependent and subjective aspects of logistic burden are typically not comprehensively collected. Home-based cancer care activities, such as administrative tasks, may have a major impact on logistic toxicity, but these activities are often less visible to clinicians and researchers. Similarly, the impact of healthcare tasks on other obligations such as family roles, daily activities, employment responsibilities, and values are not typically addressed with considering treatment burdens, but interactions with individual life circumstances likely has major implications for the subjective impact of treatment burdens [31, 32]. Therefore, based on our concept of logistic toxicity that goes beyond objective measures of time and logistic requirements of cancer care, it was our goal to gain an overarching understanding of experiences of logistic toxicity in the context of cancer. We performed in-depth interviews with individuals undergoing cancer treatment to learn about subjective experiences of logistic toxicity, how cancer-related tasks did or did not interfere with daily life, as well as patients’ ideas regarding what might be helpful to address the logistic toxicity of cancer care.

## Methods

### Study participants and recruitment

Participants in this qualitative interview study were individuals receiving cancer treatment at MHealth Fairview. MHealth Fairview is a partnership between the University of Minnesota, University of Minnesota Physicians, and Fairview Health Services which serves individuals primarily from Minnesota in the United States, along with referrals and consultations for individuals from neighboring states. We sought to recruit a clinically and demographically diverse study population, striving for representation across cancer sites, type of treatments received, gender, and age groups. English speaking individuals aged 18 years or older, currently being treated for cancer, were eligible for this study. Potentially eligible participants were identified at the time of a clinic visit by providers and approached by a research coordinator before or after a scheduled appointment. The recruitment period for this study was July 28, 2022 to November 21, 2022. They were given time to decide whether they would like to participate and were provided with the contact information of the study coordinator. All participants provided in-person paper or remote written informed consent utilizing Research Electronic Data Capture (REDCap) eConsent [33]. Demographic information—age, race and ethnicity, partner status, dependent status, income, insurance information, and education—was self-reported via survey. Cancer details including stage, treatment, and primary cancer site were obtained from electronic medical records; records were accessed for research purposes between May 24, 2023 and June 4, 2023. Identifying information was available to those collecting data and was blinded during analysis. The University of Minnesota Institutional Review Board approved this study (STUDY00015870) and all participants provide electronically signed written informed consent. The study was registered with ClinicalTrials.gov (NCT05502302).

### Interviews

We planned to complete 20 interviews. This sample size was reflective of our intention in this study of getting an overarching, exploratory understanding of subjective sources of logistic toxicity. A trained interviewer (S.H.) conducted approximately 45 minute-long interviews with eligible participants via Zoom, phone, or in-person on the University of Minnesota campus in Minneapolis, MN per participant preference. Participants confirmed their agreement to participate and for the interview to be recorded immediately prior to the start of the interview. Each session was digitally audio-recorded; those conducted via Zoom were initially transcribed using its AI Companion, manually reviewed and corrected, and presented verbatim; phone interviews were manually transcribed verbatim by two members on the research team (KB and MS). Participants received $50 for their participation.

The logistic burden research in the chronic disease management literature has highlighted the complexity of treatment burden as the concept encompasses both patient workload and experiences of healthcare tasks. Reflecting the goals of our study, we designed the interview guide to focus more on the experiential aspects of the logistic burden as the current cancer care literature has focused on objective workload aspects of the burden (Table 1). The authors drew upon theories such as the Burden of Treatment Theory [34] and the Cumulative Complexity Model, [35] as well as instruments such as the Treatment Burden Questionnaire (TBQ) [36] and the Patient Experience with Treatment and Self-management (PETS), [37] all of which emphasizes the importance of capturing patient experience, well-being, and quality of life for understanding the treatment burden. The research team and a Community Advisory Board (seven members: three oncologists, an oncology nurse, a cancer survivor, a caregiver, and a payer) reviewed the interview guide prior to the first interview. The interview questions asked what took up the most time, effort, and energy since the participant’s cancer diagnosis, how participants organize their care tasks, and how, in an ideal world, they would like to have logistic burdens addressed within cancer care.

**Table 1:** Interview questions.

Interview Questions
Being a cancer patient can require a lot of effort, for example time for appointments, waiting for providers, scans, medications, etc. We are trying to better understand everything needed in order to receive treatment, and how this effort can interfere with our everyday life and well-being. We have termed this “logistic burden of cancer care.” What comes to mind for you when you think about how your life has changed since your cancer diagnosis related to how you spend your time?
Which cancer/treatment related tasks do you think take up the most of your time, effort, and energy?
Which treatment tasks are most distressing and disruptive to your everyday life priorities?
What other treatment tasks require significant effort and/or pose significant distress?
Have you tried to record and make sense of the logistical burden of cancer treatments, i.e., the activities and trips you complete for treatment? If yes, how and why?
How has the logistical burden of treatment tasks affected your everyday life and well-being?
Have you communicated the logistic burden of cancer treatment and the related impact on your well-being to anyone? If yes, to whom and why?
In an ideal world, how would you like to communicate the logistical and well-being burden of carrying out cancer treatment tasks to your care providers, employers, family, and friends?

### Analysis

Participant interview transcripts were iteratively analyzed using an inductive thematic analysis approach [38]. Two researchers (A.C.D. and P.J.) conducted the qualitative analysis. They first each listened to the audio recordings, read the transcripts, and identified initial codes. They reviewed the codes together and then each separately reviewed the transcripts again, applying the codes to the transcripts. These codes and associated excerpts were reviewed and then grouped into themes, first by the two researchers and then with input from R.I.V. The research team and Community Advisory Board then reviewed the themes and agreed upon the final categorization of themes and subtopics. Exemplary quotes from participants are provided as appropriate.

## Results

### Participant characteristics

Of 37 identified eligible and approached individuals with cancer, 28 indicated they were willing to participate, 23 consented, and 20 (54.1%) ultimately completed the interview. The median age of participants was 55 years (range: 30–79). Just over half (11; 55.5%) identified as female, 8 (40.0%) as male, and 1 (5.0%) as non-binary (Table 2). The majority (70.0%) identified as non-Hispanic White, with three identifying as multiracial, two as non-Hispanic Black, and one as Mexican/Mexican-American/Chicano/a. Most participants were married or partnered (55%), followed by single (25%), divorced (15%), and widowed (5%). Half of participants had dependents, and employment status varied (40% employed full-time, 20% employed part-time, 20% retired, 15% not currently working, and 5% on long-term disability). Median time since diagnosis was 13 months (range: 3–202 months). The most common primary cancer sites were colorectal (30%) and breast (25%). More than half of participants had metastatic disease at the time of interview (55%). Most participants were receiving infusion chemotherapy at the time of interview (55%), followed by hormone therapy (25%), surgery in the past six months (15%), and immunotherapy (15%).

**Table 2:** Participant demographics.

Characteristic	N	Median (Range)
**Age, years**	20	55 (30–79)
**Time since diagnosis, months**	20	13 (3–202)
	**N**	**%**
**Gender Identity**		
Female	11	55.0
Male	8	40.0
Non-binary	1	5.0
**Race/Ethnicity**		
Non-Hispanic Black	2	10.0
Non-Hispanic White	14	70.0
Mexican/Mexican-American/Chicano/a	1	5.0
More than one race	3	15.0
**Highest level of education**		
High school graduate	5	25.0
Associate degree	3	15.0
College graduate	7	35.0
Graduate or professional training	5	25.0
**Relationship status**		
Married/partnered	11	55.0
Divorced/separated	3	15.0
Widowed	1	5.0
Single/never married	5	25.0
**Employment Status**		
Full time	8	40.0
Part time	4	20.0
Long-term disability	1	5.0
Retired	4	20.0
Not currently working	3	15.0
**Annual household income**		
<$20,000	3	15.0
$20,000-$49,999	4	20.0
$50,000-$99,999	5	25.0
$100,000-$149,999	3	15.0
$150,000 or more	3	15.0
Prefer not to answer	2	10.0
**Health Insurance Type**		
Employer provided	8	40.0
Spouse’s insurance	3	15.0
Medicaid/State provided insurance	5	25.0
Medicare	2	10.0
Not reported	2	10.0
**Dependents care for / support financially**		
No	10	50.0
Yes	9	45.0
Not reported	1	5.0
**Primary cancer site**		
Colorectal	6	30.0
Breast	5	25.0
Gastroesophageal	3	15.0
Pancreatobiliary	3	15.0
Prostate	1	5.0
Kidney	1	5.0
Carcinoma, unknown origin	1	5.0
**Stage at Interview**		
I	1	5.0
II	3	15.0
III	2	10.0
IV	13	65.0
Unknown	1	5.0
**Metastatic Disease at Interview**		
Yes	11	55.0
No	7	35.0
Unknown	2	10.0
**Treatment at Interview**		
Infusion chemotherapy	11	55.0
Hormone therapy	5	25.0
Surgery, past six months	3	15.0
Immunotherapy	3	15.0
Targeted therapy	2	10.0
Oral chemotherapy	2	10.0
Radiation	1	5.0
**Treatment Status at Interview**		
Front-line/up-front treatment	7	35.0
Recurrent disease	9	45.0
Maintenance	3	15.0
Other, side effect management	1	5.0

### Major themes

We identified six major themes with regards to logistic burdens of cancer care: (1) travel for care, (2) appointment time, delays, and communication challenges, (3) navigating the healthcare system and administrative tasks, (4) intersection of logistics and other burdens, (5) relationship strain, and (6) resources for managing logistic burdens.

#### 1. Travel for care

A large majority of participants identified travel for outpatient appointments as a burden of cancer care. All participants relied on transportation via car. Other methods, such as public transportation, were either not referenced or described as too time intensive. Participants living far from the cancer center described the greatest transportation burden, with several participants driving multiple hours each way for appointments. Almost every participant discussed being unable to drive themselves during at least one point in their cancer treatment process because of treatment side effects, being uncomfortable driving in the metro area, and/or difficult weather. Many relied on family and friends to drive them, which often added additional logistic burden to those individuals. Participants with limited driving support described additional barriers, such as few volunteer drivers being available in their area and having to pay for transportation services.

*“I do not drive in the metro area and neither does my daughter, who is my caregiver. So I have to depend on a volunteer driver, and they’re not always–always available.” (Female, 70–80 years)*

“*Through the winter where I really wasn’t feeling well [*…*] that was a Ub*, *an Uber ride every time*. *And yeah*, *that adds up*.*” (Male*, *40–50 years)*

In addition to burdens of traveling, several participants described significant barriers with parking at the cancer center, including cost of parking, walking distance from the parking ramp, and navigating traffic around the cancer center.

#### 2. Appointment time, delays, and communication challenges

Participants reported a wide range of experiences regarding how disruptive appointments were to their lives and wellbeing. Some felt that appointments were manageable, while others felt appointments were overwhelming and unpredictable. The latter was especially true for participants with fluctuating appointment schedules and frequencies, such as those with treatment complications requiring unplanned and additional appointments.

"*It’s when the things happen that you don’t know*, *you know*, *the unexpected um problems with your heart that require extra appointments that are annoying*. *[*…*] these extra appointments*, *these extra tests that sometimes pop up that um definitely add more stress than the usual uh schedule*. *[…] there’s a change in protocol*, *medicine*, *symptoms*, *progression*, *that is uh*, *that’s by far the worst*.*" (Female*, *60–70 years)*

Participants who needed laboratory tests to determine if it was safe for them to receive treatment that day also consistently described greater appointment burden. Participants shared that the lab was constantly behind schedule. Some, often those with greater transportation burdens such as rural participants, expressed dissatisfaction not having the option to complete labs at existing locations closer to home.

*"Sometimes treatment can take two hours longer than normal. Uh which obviously affects everything else–I get in an hour late–the labs take an extra 45 minutes–then my five hour day is seven hours and I still have to drive six hours–now it’s a 13 hours day […] I’ve had to drive here before– 90 miles–take labs and not been able to get treatment.” (Male, 30–40 years)*

Other participants also expressed frustration regarding the appointment scheduling system being more protocol-driven than patient-centered. Some mentioned feeling frustrated by redundant tasks and/or appointments not scheduled around their other life priorities.

*“I have three appointments on this coming Monday*. *And today I had to do the same e-check for all the appointments […] answer the same exact questions three times over*.*” (Male*, *40–50 years)*

“*It always seemed like they—they were not communicating appointment dates or stuff like that*. *So*, *a lot of times*, *I—I’d be going three days in a row over to [clinic] um to see three different doctors*, *instead of just being able to maybe see two in one day*.*” (Male*, *50–60 years)*

Waiting time at appointments and resulting burden was also a recurring theme. Annoyance resulting from wait times was made worse if patients felt that clinical personnel did not take wait time issues seriously.

“*Medical time is different from normal time*. *I mean—they tell you ‘well can you come 15 minutes early*,*’ so you come 15 minutes early*, *but then you wait around 45 minutes before they you know call you back there*. *[…] you see the nurse*, *she does her little triage thing*, *and then she goes “the doctor will be here in a minute” well no it’s not a minute*, *it’s 15 minutes later*.*” (Male*, *50–60 years)*

Participants felt interactions with care providers at appointments influenced their logistic burden. While participants generally praised and felt well taken care of by their care teams, some reported feeling that appointments were too short and/or providers were too busy to familiarize themselves with their situations in detail. This left some with additional burden of learning more about their condition on their own, which they felt should have been covered in their clinical appointments.

"*I’ve had to tell my story so much to people that I feel like pretty*, *I don’t know like I just feel like broken records sometimes*. *And it would be nice also if people*, *maybe just providers*, *could just like actually read my file*.*” (Non-binary*, *40–50 years)*

#### 3. Navigating the healthcare system and administrative tasks

Some participants expressed logistic barriers with the healthcare system more broadly, including administrative tasks. For example, participants found it difficult to access all of the information and resources they needed: insurance coverage and costs, provider names and contact information, proof of treatment documentation for employers, among others.

*“Finding the right doctors, making the appointments, calling the insurance company when the prescription doesn’t go through, um you know rescheduling a doctor’s appointment, um you know all, all of, all of those challenges.” (Female, 40–50 years)*

Understanding the healthcare system as a whole posed a challenge to some. One participant, who immigrated to the United States and is a non-native English speaker, reported additional barriers navigating the health system.

“*We are uh newcomers to this country*. *Hm so we don’t have enough information about hospitals*. *And also language is other difficult and this because I am bilanguage and most of the time*, *at the beginning I used the interpreter*. *Mmm but that*, *that*, *some interpreters aren’t*, *they didn’t express my idea correctly*. *That’s the difficult things for me*. *And uh sometimes we do not understand really the systems*. *How works*.*” (Female*, *30–40 years)*

Following a subsequent prompt, the same participant reported not having access to informal support systems such as community groups, neighbors, or coworkers who have had cancer, further emphasizing that all the support she had received came from the hospital only. This underscores the importance of community connectedness which those who have recently immigrated to the United States may lack.

Participants with complex care needs also described problems when needing to see multiple providers in a decentralized care environment.

“*The medical system in the United States is pretty poor at making it accessible I think*, *especially for people who have a lot of medical burden*. *It’s just so siloed and decentralized in a lot of ways*.*” (Non-Binary*, *40–50 years)*

“*Could some of these additional services that I go all over the city to receive could those someday be in an adjacent building*? *[…] so that you could maybe go from your appointments next door*.*” (Female*, *40–50 years)*

When problems were elevated to administration, some felt their concerns were not meaningfully addressed with attempts to find true solutions.

“*I’ve had numerous discussions with the lab manager*. *I’ve had I bet at least 20 conversations with the patient advocate*. *Um*, *and like I said*, *we’ve written many letters*. *Um*, *the best I’ve gotten is 2 hours of free parking–whoop de doo*.*” (Female*, *60–70 years)*

#### 4. Intersection of logistics with other burdens

Additional complexities emerged when logistics overlapped with other challenging areas of care. For example, almost every patient described how treatment side effects such as neuropathy, diabetes, fatigue, and low energy levels compounded direct time losses from cancer-related tasks, adding indirect time losses.

“*[Cancer care] probably takes up 50% of my time*, *but then you know when you do some of*, *a couple of those things a day*, *in a day*, *then you don’t have the motivation or energy to do anything else*.*” (Male*, *60–70 years)*

Logistic and financial toxicities also commonly overlapped. Patients experienced financial costs associated with their care tasks (transportation costs, parking costs, grocery delivery costs, job loss, reduction of household income, limitations on disability benefits, limitations on insurance coverage for medications and integrative medicine, copays) as well as consequences of these costs (difficulty paying for basic needs and being behind on bills). Financial uncertainty also increased the logistic workload for patients, such as not knowing if insurance covers a procedure/medication.

“*I drive 90 miles every*, *every time and I had treatments almost every week for about seven months straight*. *So it impacts uh work heavily*.*” (Male*, *30–40 years)*

Cancer forced me to leave my job. You know, so not only are you spending all this money on health care […], you’re also down half of your income. (Female, 40–50 years)

Participants who were working, had partners who were working, or had young children emphasized how cancer tasks interfered with work and family responsibilities.

“*How am I gonna get there*? *How long am I gonna be here*? *How am I gonna get home from here*?*” Um and for me personally*, *as a mom like who’s or you know especially when I have an afternoon appointment*, *I have to figure out*, *“Okay*, *who’s picking up this one from school at this time*? *Who’s picking this one from school at this time*? *Who’s you know*, *who’s doing all the things that normally I would be doing*?*” (Female*, *40–50 years)*

Conversely, protective factors that allowed for more time to manage care—such as being retired, having family support, and/or living close to the cancer center—often resulted in reduced stress from cancer logistics.

“*I’m not driving across*, *you know*, *I’m not traveling across the county lines […] It’s no big deal*, *you know*. *I uh*, *If I have an appointment*, *I go to my appointment*. *If it’s right after I get done with work*, *I’ll go right after work*. *You know*, *if I have*, *you know I make it work*.*” (Female*, *60–70 years)*

“*Because I’m*, *uh*, *I’m retired and uh I don’t have any other thing to do anyway […] If I was a full time job guy or raising a family it would really put kink into my plans*.*” (Male*, *70–80 years)*

We observed that the burdens of cancer care disproportionately affect those who are most vulnerable and have least resources to address the burdens. Overall, as explained by one participant, the cumulative burden of cancer care combined with other life tasks defined logistic toxicity:

"*This feeling of*, *like a swarm you know*. *It’s about—like one little thing by itself doesn’t make too much of a dent*. *But then it’s because I have all of these little things that are hitting me all at once*, *and like on a chronic level*, *that’s like—I feel there like is this abrasive quality that over time has like really kind of worn me out*.*[*…*] In the context of all the other things that I’m keeping track of*, *it can feel really overwhelming you know*. *So I would just say that indirectly the cancer stuff and the medical stuff puts strain on other areas of my life too*. *So it’s just you know*, *it’s kind of more like—for me*, *I think it’s all about the accumulation*. *I can’t really at this point separate out cancer from all of the other stuff*.*" (Non-binary*, *40–50 years)*

#### 5. Relationship strain

Many survivors expressed the value of logistic support from family and friends, especially in regards to transportation. Participants described how logistic burdens of their cancer care also affected friends and family members, which sometimes made them feel guilty.

“*My husband*, *uh he*, *he’d go into work um before um the store opened and get things set up for the morning*. *Then he’d come home and pick me up- we’d run down [1*.*5 hours each way] for my appointments*. *And then when we got back he was trying to go back into work*.*” (Female*, *50–60 years)*

“*I drive myself so—to not inconvenience other people cause otherwise it would be inconveniencing my family even more*.*” (Male*, *30–40 years)*

Some also described how cancer tasks threatened valued life experiences and relationships with friends and family members.

“*I barely get to leave the state to go visit my sister out in [state]*, *which she just passed away*, *but I didn’t go out there and I didn’t dare because of my health and I*, *if I missed my treatments*.*” (Male*, *70–80 years)*

“I can’t make up the time working on the weekends when I want to have my kids, and obviously I can’t be with them or wouldn’t want to be with them during the, you know, treatment days.” (Male, 30–40 years)

“*I don’t share a whole lot with [my children] because they’re really emotional about this*. *Um and um so I don’t have them to help me with a lot*.*” (Female*, *60–70)*

#### 6. Resources for managing logistic burdens

Some participants shared resources for what does or would alleviate cancer-related logistics. Participants appreciated initiatives that they felt decreased time burdens; for example, several valued home care or wished they could start or expand it.

"*Ideally I would want everything to come to my house*. *That would be my perfect situation*, *I guess; and yeah they leave with whatever they come and come and leave with whatever they need and I stay here*. *Uh yeah*, *yeah–I don’t—it’d just save me time*.*" (Male*, *40–50 years)*

“*So in the ideal world*, *everything would to be going smoothly*. *It’s like—or from—from all they give us home services actually*. *Uh because it’s hard sometimes when you have a family*, *sick kids*. *It’s too hard to take care of families*, *especially kids*. *So if the hospital provide that home services just like umm day care services*.*” (Female*, *30–40 years)*

Additional existing helpful resources included patient care coordinators, other cancer survivors, prior knowledge of or experiences with the healthcare system, shared calendars with employers, volunteer drivers, donations from community organizations, and cancer care lodging.

"*My husband was disabled and we had a lot of appointments with him*, *and I’ve done this so much*, *and then when my own health problems started coming up it just kind of fell in place*. *You kind of know what to do after a while*.*" (Female*, *70–80 years)*

“*[The impacts of logistic on well-being are] pretty negligible […] every doctor always had a patient coordinator so like everything that was scheduled was always pushed to me [*…*] a lab*, *a doctor’s appointment*, *an infusion–like all that’s taken care of behind the scenes” (Male*, *40–50 years)*

Most participants explicitly stated that they trusted, liked, and were grateful for their medical teams, with few exceptions of participants stating they felt that their care was suboptimal. Additionally, some participants cited internal or family resources that helped alleviate cancer-related stress, for example religious faith, gaining perspective and appreciation of life, or taking a proactive role in clinical decision-making and life-choices so as to live in accordance with one’s own values.

"*I went to my kids and my grandson and said*, *if I have a limited time*, *uh I want to spend as much time with–with you as possible because you are the people I love the most*. *[…] So*, *in fact*, *um*, *my my diagnosis*, *cancer hasn’t been all bad*, *because I think our family is is uh has been enriched because of the decision we made about how we deal with it*. *[…] I told my oncologist […] I will accept treatment only if I can have a quality of life*. *I refuse to be as sick as I was before*, *if this is not something we can cure*.*” (Female*, *70–80 years)*

Participants also discussed their use of technology for their cancer care. Almost all participants stated that they valued the health system’s MyChart system and associated messaging. With regards to virtual visits, there was consensus that some in-person visits are necessary; however, there was variation regarding the ideal ratio of in-person visit to virtual visit modalities. Virtual visits were helpful for many, but others said they had frequent problems with connectivity and/or usability. Additionally, participants who lived out of state expressed frustration that they had to drive to Minnesota for virtual appointments because doctors were not licensed in their home state.

In addition to existing resources and strategies for managing logistic burdens, some participants had ideas for new changes or systemic improvements. Common proposals included improved provider communication availability, increased communication between providers, early notifications of appointment delays, and appointment time consistency. A few participants mentioned easier access to provider contact information/records, prompt notification of insurance approval, better administration response, streamlined communication of medical information to employers, more intimate doctor-patient relationships, and a station at the infusion center for participants to work while receiving treatment.

## Discussion

The objective of this qualitative study was to document logistic barriers of receiving care for cancer and their reported impact on patients’ lives and wellbeing. Logistic burden research among individuals with chronic disease has expanded in the past decade, and our study adds to sparse cancer-specific research in this field. The experiences shared by participants in this study highlight multiple objective and subjective sources of logistic burden for cancer survivors and their loved ones related to travel, appointments, navigating the health system and administrative tasks, relationships, and cumulative intersectional burden.

Our findings are consistent with previous work. Transportation was a burden for almost every participant because most were unable to drive themselves during at least one point in their treatment process, which is consistent with prior studies in which transportation burdens were reported by individuals with ovarian and breast cancer [28, 31]. Cancer survivors often spend a large number of hours receiving cancer treatment [25–29], and appointment logistics also created barriers for almost all patients in this study. These costs expanded beyond appointments alone and included healthcare system barriers and administrative tasks, such as patient responsibility in communicating between care teams, which was previously reported as burdensome by survivors of ovarian cancer [31]. Another recurring theme was the impact of logistic burdens on relationships and caregivers, especially in regards to transportation, which was expected given that many patients require caregivers to attend clinic visits [11]. Our study suggests that these care burdens interact with work and life priorities and affect caregivers, relationships, and family time.

Our data suggest that the wellbeing impact of logistic burdens is often exacerbated by contexts, for example treatment side effects, financial burdens, lack of social support, and/or work or childcare responsibilities. This cumulative burden of cancer care combined with other life tasks defined subjective logistic toxicity.

Future work should focus on describing and quantifying these time costs and their influence on other life activities and responsibilities. The effect of logistic burdens differ by life circumstances, and a greater understanding may allow us to tailor treatment plans and appointments to patient needs. Increasing provider and health system awareness of patient vulnerabilities to logistic toxicity due to the compounded logistic burdens and life context may be the first step. Additionally, participants in this study had ideas for how to improve the logistic burdens of cancer care: tools they already utilized (such as using MyChart for communication with providers and homecare) as well as desired hypothetical resources (improved care coordination, more effective intradepartmental communication, and early notification of appointment delays). Some of these ideas are consistent with intervention outcomes in other chronic diseases, including care coordination programs which have improved patient outcomes and reduced family caregivers’ burdens [39, 40]. Current healthcare organizational strategies are evolving, and patient preferences and burdens may shift; for example, MyChart was utilized by a large number of our participants in this study, but this relationship may change as more healthcare systems begin to charge for some physician messaging [41].

The main strength of this study is the use of qualitative methods, which allowed for a detailed and in-depth assessment of logistic burdens and capture of personal experiences. The study population was diverse with regard to age, rurality of residence, and dependent caregiver status, providing insight into a wide range of lived experiences and life circumstances that can compound or alleviate logistic burdens of cancer care. A limitation of this study is that patients from only one academic cancer center were recruited in a city with limited public transportation options, decreasing generalizability and breadth of cancer survivor experience. The sample size of 20 interviews was likely insufficient to reach saturation with regard to nuances and contexts of diverse participants’ subjective perceptions of logistic toxicity of cancer, for example, by cancer type and among patients with limited English proficiency. While we included participants with different cancer diagnoses, burdens may be different by cancer type. We are unable to differentiate cancer site-specific burdens and did not include individuals with every primary cancer site. Only one participant in this study described navigating the healthcare system after immigrating to the United States with English as a non-primary language. Individual stories shared here demonstrate the need for more focus on experiences of those who have immigrated, refugees, gender diverse individuals, and other underrepresented and disadvantaged populations in cancer care. Lastly, employment of the interviewer by participants’ academic healthcare cancer organization may have affected participant responses.

## Conclusion

The experiences shared by participants in this study highlight multiple sources of logistic burden for survivors and their support people related to travel, appointments, navigating the health system and administrative tasks, relationships, and cumulative intersectional burden. Future work should focus on quantifying these time costs with the goal of informing interventions to reduce and mitigate the negative impacts of time and logistic burdens on patient care.

## Supporting information

S1 Checklist Human participants research checklist indicating ethical approval was granted and informed consent obtained. (DOCX)
